# *Salmonella* as an Innovative Therapeutic Antitumor Agent

**DOI:** 10.3390/ijms150814546

**Published:** 2014-08-21

**Authors:** Wen-Wei Chang, Che-Hsin Lee

**Affiliations:** 1 Department of Biomedical Sciences, College of Medical Science and Technology, Chung Shan Medical University, Taichung 402, Taiwan; E-Mail: changww@csmu.edu.tw; 2 Department of Medical Research, Chung Shan Medical University Hospital, Taichung 402, Taiwan; 3 Department of Microbiology, School of Medicine, China Medical University, Taichung 404, Taiwan

**Keywords:** *Salmonella*, tumor-targeting, antitumor, immunotherapy

## Abstract

Lack of specificity of the therapeutic agent is a primary limitation in the treatment of a tumor. The use of preferentially replicating bacteria as therapeutic agents is an innovative approach to tumor treatment. This is based on the observation that certain obligate or facultative anaerobic bacteria are capable of multiplying selectively in tumors and inhibiting their growth. Bacteria have been employed as antitumor agents that are capable of preferentially amplifying within tumors and inhibiting their growth. Moreover, bacteria-derived factors have an immune-stimulation effect. Therefore, bacteria are able to transfer therapeutic genes into the tumor cells using their infective ability. Herein, we introduce the application of bacteria for tumor therapy and focus on *Salmonella*, which have been widely used for tumor therapy. *Salmonella* have mainly been applied as gene-delivery vectors, antitumor immune activators and tumor cell death inducers. This study will not only evaluate the therapeutic efficacy of *Salmonella* for the treatment of tumor but will also elucidate the mechanisms underlying the antitumor activities mediated by *Salmonella*, which involve host immune responses and cellular molecular responses.

## 1. Introduction

Recently, general tumor treatments including surgery, radiation therapy, and chemotherapy have shown some limitations. Surgery and radiation therapy are limited to localized tumors [1], and chemotherapy may induce severe side effects. *Salmonella* are gram-negative, facultative anaerobes that are a common cause of intestinal infections. Due to substantial immunostimulation produced by *Salmonella* lipopolysaccharide (LPS) and other components, systemic infection with *Salmonella* induces proinflammatory cytokine expression and immune cell infiltration in the host [2]. Systemically administered *Salmonella* is preferentially accumulated within tumors for at least one month, forming tumor-to-normal-tissue ratios exceeding 1000–10,000 to 1 [3]. Furthermore, this accumulation is accompanied by a delay in the growth of the tumor [4,5]. *Salmonella* can grow under both aerobic and anaerobic conditions, so they are able to colonize small metastatic tumors as well as larger tumors [6,7,8]. Attenuated *Salmonella* hinder tumor growth in a broad range of human and mouse tumors [9,10]. Tumor growth is inhibited for long periods, even up to several weeks. These observations, coupled with ease of genetic manipulation, suggest that *Salmonella* are good candidates for therapeutic antitumor agents, and genetically engineered *Salmonella* have been developed to express exogenous genes, aiming to enhance their antitumor effects [2,5,11].

## 2. Host Immunity and *Salmonella*

### 2.1. Innate Immunity and Salmonella

The immune response against *Salmonella* is composed of an immediate response mediated by the innate arm of the immune system followed by antigen-specific adaptive immunity [12]. Together, these two arms of the immune system work to eradicate infection and provide long-lasting immunity and memory [13]. *Salmonella*-induced tumor inhibition occurs with alterations in myeloid cells consistent with maturation to macrophage effectors and a reduction in their suppressive capacity. The antitumor effectiveness of *Salmonella* relies on the induction of the innate immune response through the toll-like receptor-myeloid differentiation primary response gene (*TLR-MYD88*) signaling pathway [14]. This is consistent with our previous report demonstrating that *Salmonella* induce cytokine production and antitumor activities via TLR4 signaling, which may help clarify the molecular mechanism of *Salmonella*-induced host antitumor responses [15]. The increased expression of interferon (IFN)-induced chemokines in the tumor was observed during *Salmonella* treatment *in vivo*. IFN-dependent chemokines induced by *Salmonella*, such as monokine induced by IFN-γ (MIC) and IFN-inducible protein-10 (IP-10), are expected to recruit activated effector cells within the tumor. Taken together, we and other groups show that *Salmonella* significantly upregulates IFN-γ [15,16,17] and the IFN-inducible chemokines MIG and IP-10, which may be responsible for recruiting peripheral natural killer and T cells to the tumor [15].

### 2.2. T Cell Activation by Salmonella

**Figure 1:**
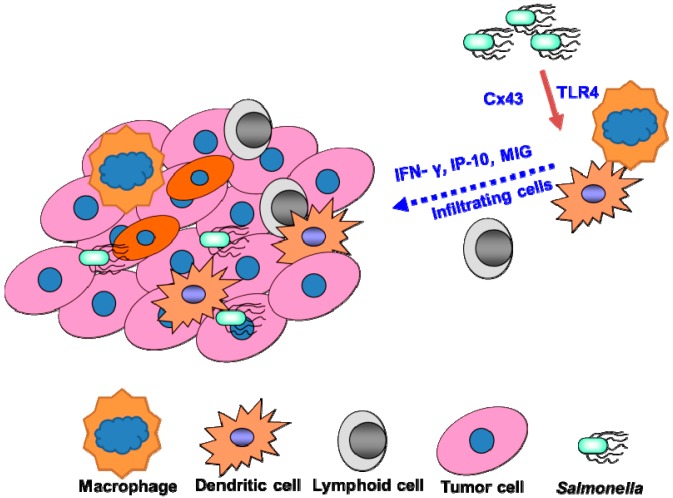
*Salmonella*-mediated stimulation of antitumor immunity. *Salmonella*-activated dendritic cell or macrophage by bacterial components, such as lipopolysaccharide (LPS), lipoteichoic acid (LTA), and flagellin. *Salmonella* inhibits tumors by increasing infiltrating cells or by cytokine expression.

*Salmonella*-induced immune responses, especially T cell activation by *Salmonella*, may include both anti-*Salmonella*-specific and tumor antigen-specific [18,19]. Rescigno *et al.* demonstrated that bacterial components, such as lipopolysaccharide (LPS), lipoteichoic acid (LTA), and flagellin, induced the expression of connexin 43 (Cx43) in tumor cells. *Salmonella* can cause melanoma cells to form gap junctions (Cx43) with adjunct immune dendritic cells. Consequently, the dendritic cells use peptides transferred from the tumor cells to stimulate T cells to recognize and kill the tumor cells at the primary site and prevent metastasis formation [19]. *Salmonella* act both locally, by recruiting T cells that inhibit tumor growth, and systemically, where *Salmonella* promote development of the immune response via the cross-presentation of tumor antigen [19]. *Salmonella* replication and lysis of tumor cells may induce cell-mediated immune responses to tumor cells by increased infiltration of CD8^+^ T cells in *Salmonella*-treated tumors [20]. The cytotoxic T cell response against tumor cells may enhance the antitumor efficacy of *Salmonella-*expressing cytokines, exhibiting an ability to modulate host immunity and inhibit tumor growth [11]. In contrast, reducing immunosuppressive cytokines in tumor can also inhibit tumor growth [21]. Binder *et al.*, reported on tumor-specific antigen delivery into tumors using *Salmonella* to increase antigen levels and generate a proinflammatory tumor microenvironment [22]. *Salmonella* is a powerful therapeutic approach to rescue dysfunctional endogenous tumor-specific CD8^+^ T cells. We used wild-type, CD4^+^ T cell-deficient and CD8^+^ T cell-deficient mice to study the role of T cells in the antitumor immune responses induced by *Salmonella*. When systemically administered into mice bearing tumors, *Salmonella* significantly inhibited tumor growth by 50%. In contrast, in CD4^+^ T cell-deficient mice, there was only a 34%–42% inhibition of tumor growth [12]. Anti-*Salmonella*-specific T cells that are induced during the infection are recruited in the flamed tumor site where they kill infected tumor cells [20]. *Salmonella* actively infects tumor cells in the presence of an expanded pool of anti-*Salmonella*-specific immune responses. During the clearances of *Salmonella*-killed tumor cells, tumor debris could be generated in large quantities, be taken up by antigen-presenting cells including macrophage and dendritic cell, and presented to naïve T cells for stimulation of tumor-specific T cells. These results suggest that tumor-targeted therapy using *Salmonella*, which exerts tumoricidal effects and stimulates T cell activities, represents a potential strategy for the treatment of tumors (Figure 1).

### 2.3. Antibodies and Salmonella

B cells play an important role in the antitumor activity mediated by *Salmonella*. Although *Salmonella* accumulated within the tumors in B cell-deficient mice, the bacterial loads of healthy organs were higher than those in wild-type mice. The inflammation cytokines and bacteremia were found in B cell-deficient mice after *Salmonella* treatment. When *Salmonella* accumulated within the tumor, B cells inhibited the dissemination of *Salmonella* to other healthy organs [23]. A major source of natural antibodies seems to be the B-1 cell subset. There is also evidence for natural antibodies with specificities for a wide range of bacterial antigens [24]. The natural antibodies produced by B cells take part in the control of *Salmonella* dissemination in tumor-bearing mice. The natural antibodies produced by B cells result in a slightly lower total number of bacteria in the tumor sites, but decreased inflammation and cytokine production in the healthy organs after systemic *Salmonella* treatment. A phase I study of attenuated *Salmonella* (VNP20009) in humans with melanoma was reported. No antitumor responses were observed and VNP20009 was detected in tumor sites in only 12.5% of patients [25]. This clinical study was not successful, which prompts the question of how these factors in the host influence the accumulation of *Salmonella* within the solid tumor. *Salmonella* have great potential as immune stimulants. However, following *Salmonella* administration, strong host immunity may develop. In addition, antibodies directed against *Salmonella* are also synthesized. The anti-*Salmonella* immune responses that exist in the host influence the tumor-targeting potential of *Salmonella* after systemic administration [13]. The loss of accumulation of *Salmonella* within the tumor contributes to the inhibition of the *Salmonella*-mediated antitumor response. These results may explain the limited accumulation of *Salmonella* within tumor sites and the non-significant antitumor response after systemic administration in clinical trials [13].

## 3.Direct Tumor-Killing Activity of *Salmonella*

**Figure 2:**
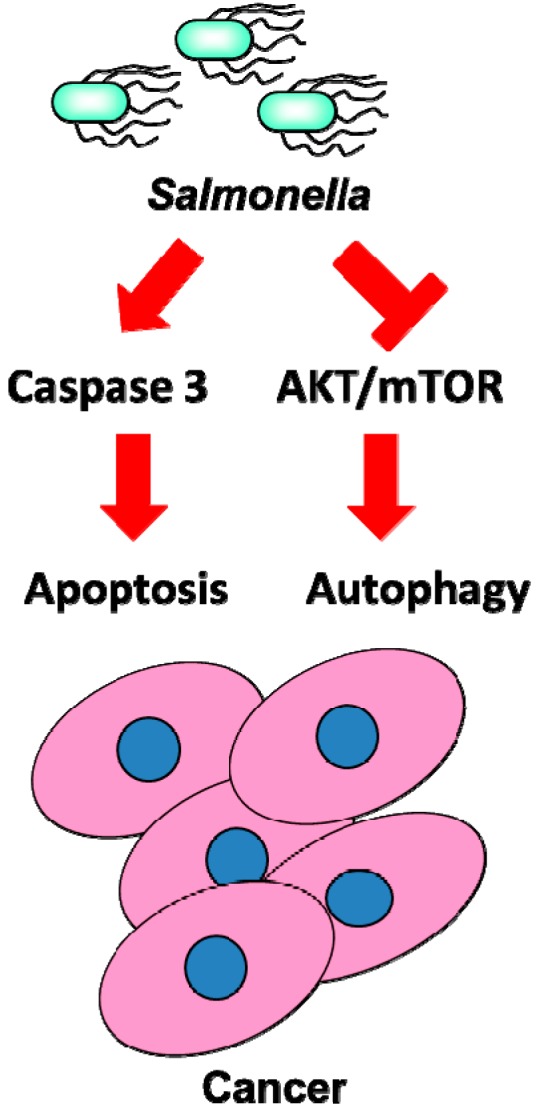
*Salmonella*-mediated cell death pathway in tumor cells. The arrow indicates that *Salmonella* induces signaling pathway. The flat indicates that *Salmonella* inhibits signaling pathway. *Salmonella* induces tumor cell death by activating caspase or down-regulating the AKT/mTOR signaling pathway.

*Salmonella* have been reported to possess antitumor activities. The mechanisms underlying their direct antitumor activities are largely unknown. Previous studies demonstrated that the induction of tumor apoptosis was correlated with *Salmonella* accumulation in the tumor sites [26]. *Salmonella* in the tumor induced apoptosis by multiple mechanisms, including competition for nutrients and stimulation of immune response [2]. Moreover, toxins from bacteria may induce the apoptosis of a tumor. As bacterial replication in tumors and subsequent lysis of tumor cells may induce cell-mediated immune responses to tumor cells, higher oncolysis could account, in part, for an increased infiltration of immune cells in tumors. The cells undergoing *Salmonella*-induced cell death exhibit heterogeneous morphological features [27]. It is clear that more than one mechanism is involved in the *Salmonella*-induced killing of cells [28]. Recently, we demonstrated that *Salmonella* may induce cell death via apoptosis and autophagic pathways by using an autophagy inhibitor (3-methyladenine) and an apoptosis inhibitor (Z-VAD-FMK). Autophagy is involved in the cell-defense elimination of bacteria [29]. The signaling pathways leading to activation of bacteria-induced autophagy in tumor cells remain to be elucidated. The AKT/mTOR/p70S6K signaling pathway negatively regulates autophagy. We determined that the levels of phosphorylated AKT, mTOR, and p70S6K were significantly decreased in *Salmonella*-treated tumor cells [30]. These results showed that *Salmonella* can induce autophagic activities in addition to caspase-dependent cell death in tumor cells (Figure 2). Autophagy may co-occur with apoptosis in tumor cells exposed to *Salmonella*. Furthermore, at later stages of infection, autophagy may partially participate in the execution of tumor cell death by enhancing apoptosis. When apoptosis is blocked, infected tumor cells undergo increased autophagy. These data suggest that *Salmonella* treatment efficiently induces both autophagy and apoptosis, which partner to induce cell death cooperatively by modifying beclin-1 and caspase expression. Apoptosis and autophagy existed crosstalk between two pathways. *Salmonella i*nduced both apoptosis and autophagy. Both apoptosis and autophagy cooperate to lead to tumor cell death after *Salmonella* infection.

## 4. Combination Therapy with *Salmonella*

Avoiding side effects has emerged as a key issue in the development of safe *Salmonella* therapy approaches. Weiss *et al.*, suggested that *Salmonella* can be readily controlled by systemic administration of antibiotics [31]. Recently, the combination of a traditional Chinese medicine herbal mixture and *Salmonella* enabled the bacteria to be safely administered at a high dose with increased treatment efficacy and reduced toxicity compared to monotherapy with *Salmonella* [32]. These results will contribute to increasing the safety and the therapeutic potential of tumor-targeting *Salmonella*. Previously, we exploited *Salmonella* as a single tumor-targeting antitumor agent and as part of a combination therapy with chemotherapy for mice bearing tumors [3]. Our results indicated that the combination of *Salmonella* and cisplatin exerts additive therapeutic effects in delaying tumor growth and prolonging the survival of the tumor-bearing mice [3]. Recently, we further found that *Salmonella* not only cause increased intercellular communication between tumor cells due to Cx43 up-regulation, but also increased mitogen-activated protein kinase (MAPK) signaling pathways [33]. *Salmonella* presumably enhance the response to chemotherapy agents by increasing the passage of these drugs between neighboring tumor cells. The combination of *Salmonella* with 5-fluorouracil (5-FU) improved the antitumor efficacy compared with 5-FU monotherapy in pancreatic stem-like cells [34]. Chemotherapeutic agents that enhance antitumor efficacy would involve inhibiting the viable rim of tumor growth or modifying the tumor matrix, which would facilitate the motility and penetration of *Salmonella* [3]. By taking advantage of the tumoricidal effect of *Salmonella* and the pleiotropic activities of chemotherapeutic agents, *Salmonella* in combination with chemotherapy appears to hold promise for the treatment of solid tumors.

## 5. Modification of *Salmonella*

The anti-*Salmonella* immune responses that exist in the host influence the tumor-targeting potential of *Salmonella* after systemic administration. The loss of accumulation of *Salmonella* within the tumor contributes to the inhibition of the *Salmonella*-mediated antitumor response. The bacterial cell wall is negatively charged due to the presence of either teichoic acid in gram-positive bacteria or LPS in gram-negative bacteria. Positively charged polyelectrolytes can bond to negatively charged *Salmonella* surfaces to form thin films that circumvent preexisting immunity against *Salmonella* [35]. The encapsulation of *Salmonella* in polymer reduces the antigenicity of *Salmonella* following *in vivo* delivery. The polymer-modified *Salmonella* bacteria not only resist the binding of neutralizing antibodies in peripheral blood but also replicate in tumor sites, which is believed to stimulate non-specific antitumor immunity. In other words, polymer shields *Salmonella* from neutralizing antibodies. Polymer-modified *Salmonella* also display lower toxicity and improved efficacy and safety. Mice treated with *Salmonella* had a 9% lower average body weight compared with mice treated with PBS. In contrast, the body weights of mice treated with polymer-modified *Salmonella* were not significantly decreased. After *Salmonella* and polymer-modified *Salmonella* treatment, the levels of inflammatory cytokines including interleukin-1β (IL-1β) and tumor necrosis factor-α (TNF-α) were measured in the sera. The induction of inflammation cytokines (*i.e.*, IL-1β and TNF-α) in mice treated with *Salmonella* was increased by 1.5–4.5-fold compared with induction by polymer-modified *Salmonella* treatment [35]. Polymer also can provide a useful platform for the chemical modification of *Salmonella*, perhaps allowing other chemotherapeutic drugs to bind to tumor-targeting *Salmonella*. Recently, the masking of *Salmonella* with a polymer and DNA resulted in the tumor-targeting gene transfer of *Salmonella* following *in vivo* delivery [36]. These results suggest that tumor-targeted gene therapy using polymer-modified *Salmonella* carrying the therapeutic gene, which exerts antitumor activities, represents a promising strategy for the treatment of tumors.

## 6. Conclusions

In this study, we have described the utility of *Salmonella* for tumor treatment. However, we believe that no powerful therapeutic agents are available to eradicate various types of tumor. *Salmonella* is a useful antitumor agent due to its tumor-targeting potential, antitumor capability, and ability to deliver therapeutic genes. Thus, *Salmonella*-mediated tumor therapy may have an important role in tumor treatment. These results will contribute to new research that gives thought to the complex interactions between bacteria and host immunity in order to maximize the chances of therapeutic success.
